# Incorporation of a Comorbidity Index in Treatment Decisions for Elderly AML Patients Can Lead to Better Disease Management—A Single-Center Experience

**DOI:** 10.3390/hematolrep16040074

**Published:** 2024-12-03

**Authors:** Cristina Negotei, Iuliana Mitu, Silvana Angelescu, Florentina Gradinaru, Cristina Mambet, Oana Stanca, Mihai-Emilian Lapadat, Cristian Barta, Georgian Halcu, Carmen Saguna, Aurora Arghir, Mihaela Sorina Papuc, Andrei Turbatu, Nicoleta Mariana Berbec, Andrei Colita

**Affiliations:** 1 Department of Hematology, “Carol Davila” University of Medicine and Pharmacy, 050474 Bucharest, Romania; cristina.negotei@umfcd.ro (C.N.); silvana.angelescu@umfcd.ro (S.A.); cristina.mambet@umfcd.ro (C.M.); oana.stanca@umfcd.ro (O.S.); mihai-emilian.lapadat@umfcd.ro (M.-E.L.); cristian.barta@umfcd.ro (C.B.); carmen.saguna@umfcd.ro (C.S.); andrei.turbatu@umfcd.ro (A.T.); andrei.colita@umfcd.ro (A.C.); 2 Clinic of Hematology, Coltea Clinical Hospital, 030171 Bucharest, Romania; iuliana.mitu@rez.umfcd.ro (I.M.); florigrad@yahoo.com (F.G.); georgian.halcu@drd.umfcd.ro (G.H.); aura_arghir@yahoo.com (A.A.); ela_papuc@yahoo.com (M.S.P.)

**Keywords:** AML, elderly, ECOG, HCT-CI, CCI, OS

## Abstract

**Introduction:** Acute myeloid leukemia (AML) is a form of cancer originating from precursor cells within the bone marrow. Elderly patients with acute leukemia require a personalized approach, considering age, performance status, and comorbidities, to determine suitability for intensive treatment. **Methods:** We studied the results of intense chemotherapy in 46 elderly, fit individuals with AML at a cancer center in Romania from January 2017 to December 2023. **Results:** The study involved a cohort of 46 patients, including 22 men and 24 women. The research indicated that 89.1% of the patients were diagnosed with de novo acute leukemia. Most patients had an ECOG score of 0–1, with one patient scoring ≥2. HCT-CI > 4 was found in 21 patients (45.7%), while CCI > 4 was present in 38 patients (82.6%). After the induction phase, 25 patients (54.3%) achieved complete remission (CR); the relapse rate was 56.8%. Upon completion of the study, nine individuals (19.6%) were still alive. The overall survival duration ranged from 0 to 33 months, with a median survival time of 8 months (CI 5.0–11.0). **Conclusions:** When considering treatment options for elderly patients, the Eastern Cooperative Oncology Group (ECOG) Performance Status, as well as comorbidity indices such as the Hematopoietic Cell Transplantation-Specific Comorbidity Index (HCT-CI) and the Charlson Comorbidity Index (CCI), have shown promising results in the literature, indicating their relevance in the decision-making process.

## 1. Introduction

Acute leukemia is a neoplastic disorder of hematopoietic stem cells marked by genetic code alterations that disrupt hematopoiesis, leading to an uncontrolled proliferation of immature clones replacing the normal polyclonal population in the marrow. Leukemia is commonly categorized based on the specific type of stem cell that has undergone a malignant transformation, which can be either lymphoid or myeloid [1]. Acute myeloid leukemia (AML) predominantly impacts the elderly, with the median reported age at diagnosis being approximately 70 years [2].

Managing elderly patients diagnosed with acute leukemia requires a comprehensive and individualized approach. Age, performance status, and comorbidities are commonly used to determine fitness for intensive treatment [3]. In clinical studies, it has been demonstrated that elderly patients exhibit a less favorable prognosis compared to their younger counterparts. Specifically, individuals aged 60 years or older tend to face an unfavorable prognostic outlook. The data highlight the significantly lower long-term survival rates among elderly patients, typically 9–10% at 3 years, compared to nearly 50% in younger patients [4,5]. When assessing physical fitness, age alone should not be used to determine which patients are unfit. In clinical practice, there are multiple instances where younger patients with comorbidities exhibit a poorer performance status compared to older individuals with better health. This introduces an important question: Does a person’s biological age really correspond to their chronological age? It is evident that age alone should not be the sole factor in determining fitness or treatment [6]. However, studies have shown improved outcomes in older and traditionally unfit patients, accentuating the significance of considering other factors that can predict treatment outcomes [3].

The Eastern Cooperative Oncology Group (ECOG) Performance Status is a widely used method for evaluating patients’ performance and activity levels in relation to their illness. It provides a standardized way to assess how a patient’s disease is affecting their daily functioning and physical abilities. The ECOG Performance Status scale ranges from 0 (fully active) to 5 (deceased), offering a clear picture of the patient’s overall condition. Research has found that a low ECOG is linked to lower complete response rates, increased two-month mortality, and reduced overall survival. Real-life statistics have demonstrated that ECOG influences both survival outcomes and achieving complete remission, in addition to age [6,7].

The Charlson Comorbidity Index (CCI) is a scoring system designed to assess the impact of comorbid conditions on patients’ health and predict their 10-year survival. By assigning a score to various pre-existing medical conditions such as heart disease, diabetes, and kidney disease, the CCI helps healthcare providers better understand the overall health status of a patient and make informed decisions about their care [7].

The Hematopoietic Cell Transplantation Comorbidity Index (HCT-CI) is used to make forecasts and anticipate early mortality in older patients aged 60 and above. An elevated HCT-CI score means lower survival for adult patients with acute leukemia (AL). This shows how important it is to closely monitor and give personalized medical care to these patients [8].

In the evaluation of acute leukemia’s prognosis, the cytogenetic and molecular examination holds great significance, categorizing AML into favorable, intermediate, and adverse risk groups [9].

In both AML and ALL, in cases where there is a lack of cytogenetic information due to karyotype failure or the absence of cytogenetic testing, adult patients may face potential challenges in receiving optimal treatment customized to their specific risk profiles. This lack of critical genetic data could lead to suboptimal therapy choices, potentially affecting the overall treatment outcomes for these individuals [10].

Regarding AML treatment, it is important to remember that despite the challenges, there is hope. With a 5-year survival rate of around 30%, some individuals can overcome this disease [11]. It is widely known that intensive chemotherapy (IC) can be a helpful therapy option for carefully chosen older patients.

For fit patients without significant comorbidities, the use of intensive chemotherapy has displayed a capacity to increase overall survival (OS) and response rates in individuals aged 70 years or older [12]. The use of low-intensity therapy, in combination with novel targeted therapy (bcl-2 inhibitors) or just monotherapy, offers better survival rates for older patients and those with comorbidities. This approach reduces the significant toxicity caused by IC [13].

The purpose of this study is to analyze the clinical and paraclinical characteristics and results of elderly patients with AL who were treated with IC despite the presence of innovative therapies.

## 2. Materials and Methods

We conducted a retrospective analysis to evaluate the clinical outcomes of elderly, medically fit patients who were diagnosed with AML, including both de novo and secondary cases. Patients received intensive chemotherapy at Coltea Clinical Hospital’s Hematology Department in Bucharest, Romania. The study specifically focused on patients diagnosed with AML between 1 January 2017 and 31 December 2023, with the aim of providing a detailed examination of their treatment outcomes and overall prognosis. Patients who were diagnosed with acute promyelocytic leukemia (APL), biphenotypic leukemia, blastic plasmacytoid dendritic cell neoplasm, or those solely diagnosed in our facility and subsequently treated elsewhere were excluded from our study. All patients diagnosed with AML over the age of 65 who underwent intensive chemotherapy were included. We documented their age, gender, date of arrival at our clinic, Eastern Cooperative Oncology Group (ECOG) Performance Status, Hematopoietic Cell Transplantation Comorbidity Index (HCTCI), Charlson Comorbidity Index (CCI), comorbidities, left ventricular ejection fraction, type of AML, hemoglobin level, platelet count, leukocytes, blasts in bone marrow, molecular biology, karyotype, treatment and response to treatment, disease and treatment complications, cause of death, and date of death. The analysis process involved reviewing patients’ medical records and entering the data into an Excel spreadsheet for further examination.

Treatment regimens utilized: the first line was represented by cytarabine with anthracycline, followed by intermediate-dose cytarabine. Patients with FMS-like tyrosine kinase 3 (FLT3) gene mutations, like internal tandem duplication (FLT3-ITD) or tyrosine kinase domain (FLT3-TKD), received 3 + 7 induction therapy and high-dose Cytarabine consolidation along with Midostaurin as the new standard treatment [14]. The use of MEC (Mitoxantrone, Etoposide Cytarabine), EMA (Etoposide, Mitoxantrone, Cytarabine), and S-HAM (sequential high-dose Cytarabine and Mitoxantrone) was common in the second line of treatment. When it comes to third-line treatment, LDAC-type treatment (low-dose Cytarabine) was predominantly administered, followed by the use of 6-mercaptopurine, PRET-type treatment (Etoposide, 6-thioguanine, Prednisolone), and S-HAM.

The statistical analyses were conducted using SPSS version 26. We used the Kaplan–Meier analysis to determine the average overall survival time and the average survival time without disease progression (PFS) for the entire study group without any adjustments. The median value was used to estimate the average survival time. For a comparative analysis of survival time, we used the Kaplan–Meier comparative analysis with the log-rank (Mantel–Cox) test based on demographic risk factors and other clinical characteristics. To assess the impact of a risk factor on survival, we calculated the hazard ratio (HR) using Cox proportional hazard regression analysis. We considered a *p*-value of less than 0.05 to be statistically significant.

## 3. Results and Discussion

Between January 2017 and December 2023, our cancer center provided intensive treatment to 46 patients diagnosed with AML. The diagnostic process included bone marrow cytological examination, bone marrow flow cytometry, molecular biology, and karyotype. We classified patients into different risk categories based on the European LeukemiaNet (ELN) 2017 guidelines [15]. When karyotyping information was not available, we relied entirely on molecular biology to determine the risk category for the patients.

The cohort consisted of 46 patients, which included 22 males and 24 females in terms of gender distribution. The study found that 89.1% of patients had de novo leukemia, while the rest had secondary leukemia after myelodysplasia or treatment-related.

Patients were stratified by risk group based on cytogenetic and molecular biology examinations, where applicable. A total of 39 individuals (84.8%) received a molecular biology examination; 92.3% (36 individuals) were tested for FLT3 mutations. Among the tested cohort, eight individuals (20.5%) displayed the ITD mutation, while two patients (5.1%) exhibited the FLT3 TKD mutation. Additionally, two patients tested positive for CBFB-MYH11 (16.7%), underscoring the importance of comprehensive mutation analysis in patient management and treatment strategies. Furthermore, the patients were tested for additional mutations, including NPM1, KMT2A and RUNX1-RUNX1T1, and the results were negative in each case. Only 24 (52.1%) underwent a cytogenetic test. Among those who had the cytogenetic test, 20 (83.3%) were found to have a normal karyotype.

The research group included a nearly equal number of males (24) and females (22), with a *p*-value of 0.768 > 0.10. Most patients, 43 out of 46, were in the 65–74 age range (*p* < 0.001). A total of 41 out of 46 patients (*p* < 0.001) were diagnosed with de novo acute myeloid leukemia (AML). Additionally, of the 24 patients assessed, 20 exhibited a normal karyotype and were classified in the favorable risk category (*p* < 0.001).

From the point of view of molecular biology mutations, the set contains different proportions related to their presence or absence (14/25; *p* = 0.078 < 0.10).

All patients had comorbidities, with 30 out of 46 individuals having more than three (*p* < 0.050); all patients had normal LVEF. A significantly higher ratio of patients had leukocytes > 10,000 (29/46; *p* < 0.001), platelets < 100,000 (34/46; *p* < 0.001), and a percentage of blasts > 50% (33/46; *p* < 0.001).

Cox proportional hazards regression analysis found no significant link between survival and the percentage of blasts. The analysis showed a chi-square value of 0.48 (df = 1) with a *p*-value of 0.489. The parameter estimate was −0.26, with a standard error of 0.50, leading to a Wald statistic of 0.50 (df = 1) and a *p*-value of 0.504. This suggests that people with more than 50% blasts have a 23% lower risk of death (hazard ratio = 0.77; 95% CI: 0.38–1.58). However, this finding is not statistically significant.

An increased proportion of individuals had a CCI greater than 4 (38 out of 46; *p* < 0.001). For the HCT-CI, while more scores exceeding 4 were noted (21 out of 46; *p* < 0.001), the weights of the intervals 0–2 and ≥3 were approximately equal (19 out of 27; *p* > 0.10). In the case of ECOG, only one patient presented an ECOG score ≥ 2 (Table 1).

As part of the induction therapy, 29 (63.04%) patients received the standard “3 + 7” regimen, (21.73%) received “3 + 7” along with an FLT3 inhibitor (e.g., midostaurin), and 17 (36.95%) received the “5 + 2” regimen.

Seven individuals (15.2% of the total) died during the induction phase. Following the induction phase, 25 patients (54.3%) achieved complete remission (CR) and moved on to the consolidation phase. One patient (2.2%) achieved partial remission (PR), while thirteen patients (28.3%) did not respond to the initial course of induction and were considered refractory.

In the second line of treatment, the most often used regimens were MEC (84.3%), EMA (12.5%), and SHAM (3.12%). In the context of third-line treatment, the predominant strategy involved the administration of LDAC-type treatment (46.6%), with subsequent utilization of 6-mercaptopurine (26.6%), PRET-type treatment (20%), and S-HAM (6.66%). Table 2 displays the lines of treatment along with their respective response rates.

In our analysis, the relapse rate was 56.8%. Specifically, 14 patients (66.7%) experienced relapse within a six-month period, while 8 patients (25%) encountered a subsequent relapse. The study results indicate that 56.5% of the patients experienced a survival period exceeding 6 months, with 32.6% surpassing the 12-month mark.

In the cohort under investigation, 21 out of 46 patients (45.65%) attained red blood cell transfusion independence (RBC-TI), while 19 out of 46 patients (41.30%) achieved platelet transfusion independence consequent to the treatment.

In the analysis of the first line of treatment, the complete response (CR) rate was found to be significantly higher (25 out of 46, *p* < 0.001) compared to the rates of non-response (13 out of 46) and death (7 out of 46), except the cases with CRi and PR that have a very low ratio.

For the second line of treatment, the response is notably heterogeneous. The rates of response categories—such as RC at 9 out of 32, non-response at 12 out of 32, and death at 7 out of 32—show no significant differences (*p* > 0.10). This variability is also evident in the third line of treatment.

More than half of the patients who survived the first line of treatment (21 out of 37, or 59.5%) experienced at least one relapse. This relapse rate is not significantly different from that of patients who did not experience a relapse (*p* > 0.10).

From an overall survival perspective, some heterogeneity was observed, as the proportions assigned to the three temporal categories did not differ significantly (*p* > 0.10).

According to Table 3, all patients in the study experienced complications; the most common were hematological ones. Among the non-hematological complications, 40 patients (87%) had bacterial infections (*p* < 0.010). Cardiovascular events occurred in 26 of 46 cases and hemorrhagic complications in 28 of 46 cases, both approximately 50% (*p* > 0.10). Additionally, 13 patients (28.3%) contracted the SARS-CoV-2 virus, with a *p*-value of less than 0.001, and 3 of them died.

In our cohort of patients, nine individuals (19.6%) were still alive at the end of the study. The primary cause of mortality was attributed to bacterial infection (38.9%), followed by hemorrhagic events (30.6%). Within the first 60 days after diagnosis, nine patients died.

Table 4 presents the results of the Kaplan-Meier analysis for OS and the chi-square test.

**Figure 1:**
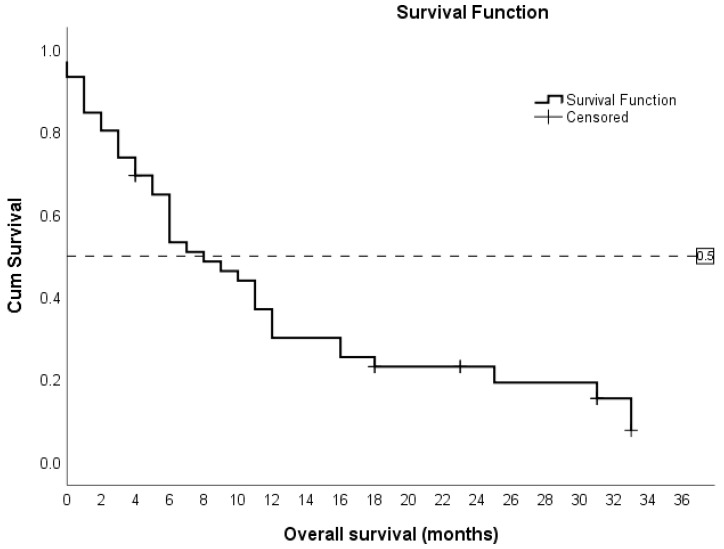
The median overall survival in patients treated with IC was 8 months.

The study indicated that the overall survival duration ranged from 0 to 33 months, with a median survival time of 8 months (CI 5.0–11.0), as Figure 1 exhibits. At 36 months, 9 out of 46 patients (19.6%) were alive, while 37 (80.4%) had died.

A log-rank test (Mantel–Cox) was conducted to assess the disparity in average Kaplan–Meier survival times between Line 1 response categories (RC and no RC). The findings reveal statistically significant and distinct survival distributions for the two response categories, with χ^2^(1) =9.89 (*p* < 0.005).

The Cox proportional hazards regression analysis showed a significant correlation between survival and complete response (CR) duration after first-line treatment, with a *p*-value of 0.022. The estimated parameter was −1.22, indicating a reduction in the expected risk of death by 0.30 times for those with a CR duration greater than six months compared to those with a duration less than six months. The statistical significance of this result was confirmed by a Wald test with a *p*-value of 0.028. The hazard ratio (HR) was 0.30, with a 95% confidence interval of 0.10–0.88.

The log-rank (Mantel–Cox) test was used to compare the average Kaplan–Meier survival times for ECOG categories 0–1 and 2–4. The results showed that the survival times for these two categories are significantly different. Specifically, the difference was found to be statistically significant, with a χ^2^ value of 14.33 and *p*-value < 0.001. It is important to note that the difference cannot be considered because the ECOG 2–4 category only had one patient who did not survive for more than one month.

**Figure 2:**
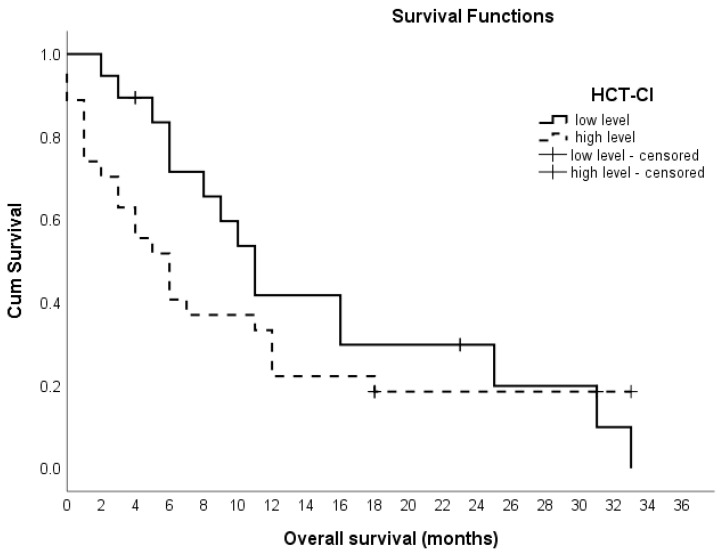
The median OS based on the HCT-CI level. For HCT-CI 0–2, the median OS is 11 months; for HCT-CI 3–4, the median OS is 6 months.

According to the Kaplan–Meier test, there are differences in the average survival tendency based on the HCT-CI level (0–2 vs. 2–4+). Specifically, the average survival trend for individuals with a low HCT-CI level (median = 11.0 months) is higher than that for individuals with a high HCT-CI level (3–4+), where the median is only 6.0 months, as shown in Figure 2. After conducting an analysis and comparison of gender-based average survival trends, it was found that in the context of HCT-CI (3–4+), women face smaller survival rates, with a threefold higher anticipated risk of mortality in comparison to men. Moreover, the risk of death is three times higher in patients aged over 75 compared to those aged 65–75.

Table 5 presents the results of the Cox regression analysis, providing an overview of the analyzed survival curves.

**Figure 3:**
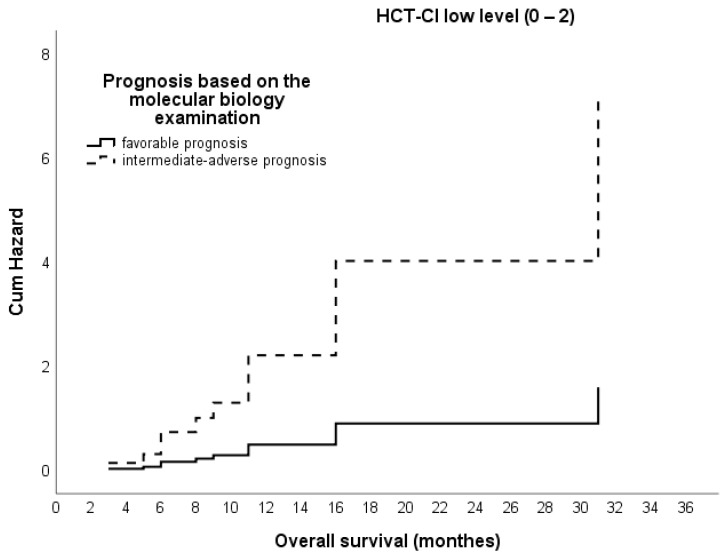
Cumulative hazard of overall survival based on Cox proportional hazards model according to prognosis based on the molecular biology examination and HCT-CI 0–2: low-level HCT-CI and an intermediate–adverse prognosis increase the risk of death by 4.5 times compared to those with a favorable prognosis.

The result obtained from the Cox proportional hazards regression analysis does not support the assumption that survival is associated with the prognosis based on the result of the cytogenetic test (χ^2^ (df = 1) = 1.24, *p* = 0.265). The estimated parameter of 0.70 (B = 0.70; SE = 0.59) does not reach statistical significance (Wald(df = 1) = 1.39, *p* = 0.239). On the other hand, regarding the association of survival with the prognosis based on the molecular biology examination in the case of low-level HCT-CI, the estimated parameter of 1.5 is statistically significant. Based on the positive value, we can assume that in the intermediate–adverse prognosis, there is an expected risk of death that is approximately 4.5 times higher than in the case of those with a favorable prognosis, as can be see in Figure 3.

The regression analysis did not support a survival association between the chemotherapy or radiotherapy administered before the diagnosis of acute leukemia in cases with a high level of HCT-CI (3–≥4).

When choosing the right therapy for elderly patients, it is also important to consider the CCI score. In our study, the results from the Cox proportional hazards regression analysis suggest a correlation between survival and age difference for patients with a high CCI score (>4). The estimated parameter of 1.55 is statistically significant. Based on this positive value, we can infer that patients over 75 years old have a risk of death approximately 4.5 times higher than those aged between 65 and 74 years.

**Figure 4:**
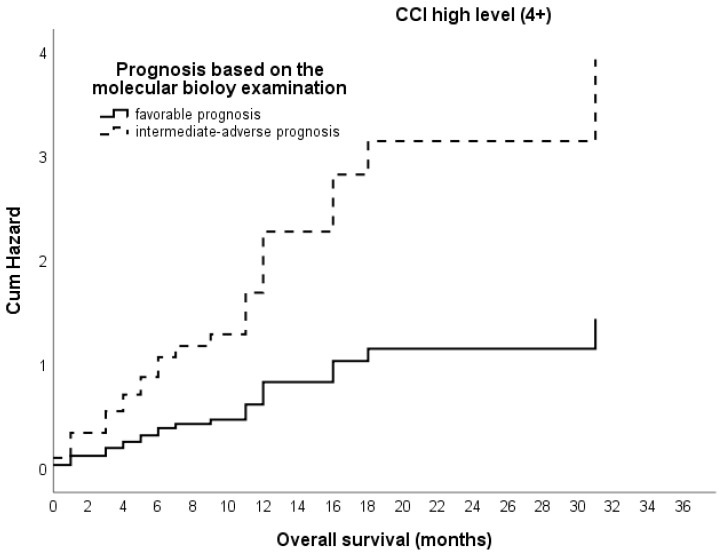
Cumulative hazard of overall survival based on Cox proportional hazards model according to prognosis based on the molecular biology examination and CCI > 4: a high CCI score (>4) and an adverse prognosis increase the risk of death by 2.7 times compared to those with a favorable prognosis.

The CCI score and the HCT-CI score did not have enough data values for the cytogenetic examination. However, when looking at the association of survival with the prognosis based on the molecular biology examination for a high-level CCI (>4), the estimated parameter is 1.0. This suggests that for patients with an intermediate-to-adverse prognosis, the expected risk of death is 2.7 times higher compared to those with a favorable prognosis. This is indicated in Figure 4.

Unlike the HCT-CI score, in the case of a high CCI score (>4), the results obtained in the regression analysis allowed the assumption of the association of survival with previous chemotherapy, thus resulting in an expected risk of death three times higher than in the case of those who were not exposed to this type of treatment.

**Figure 5:**
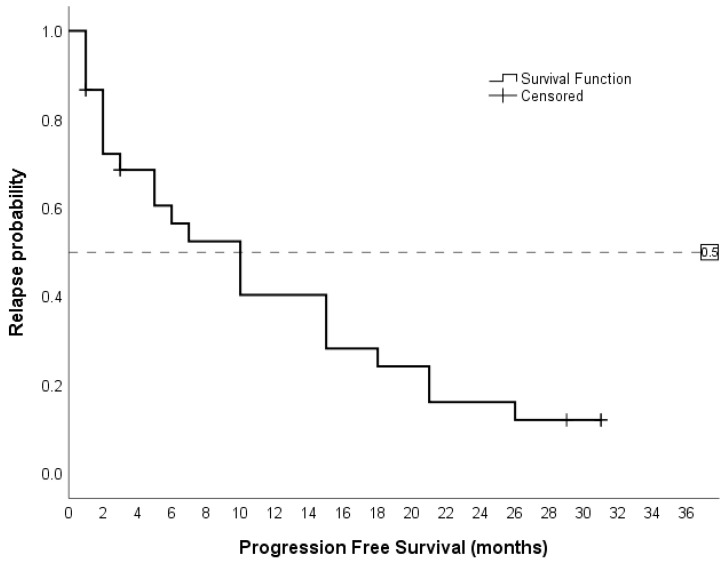
The median PFS in patients who achieved at least CR, PR, or RCi was 10 months.

In the PFS calculation, only 30 patients were evaluated. These patients achieved at least one of the following: complete remission, partial remission, or complete remission without hematological recovery. Seven patients died after the first-line treatment, and nine showed no response. In the Kaplan–Meier analysis, the median survival time was found to be 10 months (95% CI: 5.30–14.70), as stated in Figure 5.

We used a Cox proportional hazards regression analysis to study how progression-free survival (PFS) is affected by molecular biology and cytogenetic tests. We divided the tests into “favorable” and “intermediate–adverse” categories. This allowed us to see how the risk of relapse differed based on these factors. Our analysis showed a significant connection, indicating that patients with an abnormal karyotype have a 5.9 times higher risk of relapse and those with molecular anomalies have a 15 times higher risk. The analysis conducted to assess the difference in relapse risks based on the HCT-CI level (0–2, 3–4+) and CCI score did not show any significant association.

Cox proportional hazards regression analysis found no significant link between survival without disease progression and whether the cases were de novo or secondary. The analysis showed a parameter estimate of 0.84, with a standard error of 1.03. The Wald statistic was 0.67, and the *p*-value was 0.414. This suggests that patients with de novo cases had a 1.32-fold increase in progression-free survival. However, this finding is not statistically significant, with a hazard ratio of 2.32 and a 95% confidence interval of 0.31 to 17.43.

The study used Cox regression analysis to examine the survival without disease progression for patients exposed to a toxic environment or previous chemotherapy or radiotherapy. The analysis found that the highest risk of relapse was associated with radiotherapy (5.9 times higher), followed by chemotherapy (3.7 times higher) and exposure to a toxic environment (3 times higher).

## 4. Conclusions

This study has provided valuable insights indicating that the overall survival duration ranged from 0 to 33 months, with a median survival time of 8 months (CI 5.0–11.0). Compared to data in the literature, where the CR after the first-line treatment is 60–70%, our study shows a CR of 54.3% [2]; nine patients were still alive at the end of the study.

It is noteworthy that patients over 75 face a risk of death approximately three times higher compared to those aged 65–75; more specifically, according to the CCI score, the risk of death increases by up to 4.5 times for this age category. The Cox proportional hazards regression analysis revealed a statistically significant relationship between survival and complete response (CR) duration after first-line treatment. Specifically, individuals with a low HCT-CI level (median = 11.0 months) exhibited a higher average survival time compared to those with a high HCT-CI level (3–4+), where the median was only 6.0 months.

No statistically significant relationship was found regarding the prognosis based on the cytogenetic examination and the duration of global survival. This underscores the need for further research in this specific area. When we look at the link between survival and molecular biology test results for low-level HCT-CI, we find a significant estimated parameter of 1.5. This indicates that for individuals with an intermediate-to-adverse prognosis, the anticipated risk of mortality is approximately 4.5 times greater than for those with a favorable prognosis.

Regarding PFS, our analysis uncovered a significant connection indicating that patients with abnormal karyotypes have a 5.9 times higher risk of relapse and those with molecular anomalies have a 15 times higher risk. The analysis of the difference in relapse risks based on the HCT-CI level (0–2, 3–4+) and CCI score did not show any significant association.

It is essential that the concept of AML in elderly patients as an incurable illness is changed. As we may know, many patients are not properly evaluated at diagnosis. Based on the data presented, it is evident that age should not be considered the primary determinant when selecting intensive chemotherapy. We need to focus on studying and implementing individualized management based on comorbidity scores and defined subgroups identified through cytogenetic and molecular biology exams to provide optimal care for these patients. In the current age of numerous novel therapies, it is important to conduct further studies to reevaluate the necessity and effectiveness of intensive chemotherapy.

## Figures and Tables

**Table 1:** Characteristics of patients diagnosed with AML who had undergone intensive chemotherapy treatment.

Variable		Number of Patients	Percentage (%)	Chi-Square Test of Proportion		
				χ^2^	A	*p*
Age (years)	65–74	43	93.5	34.78	1	<0.001
	>75	3	6.5			
Gender	Male	24	52.2	0.09	1	0.768
	Female	22	47.8			
AML classification	De novo	41	89.1	28.17	1	<0.001
	Secondary	5	10.9			
Karyotype	Normal	20	83.3	10.67	1	<0.001
	Abnormal	4	16.7			
Risk stratification	Favorable	20	83.3	27.00	2	<0.001
	Intermediate	2	8.3			
	Adverse	2	8.3			
Molecular biology	Yes	39	84.8	22.26	1	<0.001
	No	7	15.2			
The presence of mutations	Yes	14	35.9	3.10	1	0.078
	No	25	64.1			
Risk stratification	Favorable	27	69.2	23.23	2	<0.001
	Intermediate	8	20.5			
	Adverse	4	10.3			
ECOG	0–1	45	97.8	42.09	1	<0.001
	2–4	1	2.2			
CCI	2	0	0	19.57	1	<0.001
	3	0	0			
	4	8	17.4			
	>4	38	82.6			
HCT-CI	0	7	15.2	12.26	3	<0.010
	1–2	12	26.1			
	3–4	6	13.0			
	>4	21	45.7			
Number of comorbidities	<3	16	34.8	4.26	1	<0.050
	>3	30	65.2			
LVEF	Normal	46	100			
	Abnormal	0	0			
Leukocyte (mmc)	<4000	11	23.9	19.09	2	<0.001
	4000–10,000	6	13.0			
	>10,000	29	63.0			
Hemoglobin (g/dL)	<8	28	60.9	2.17	1	0.140
	>8	18	39.1			
Platelets (mmc)	<20,000	10	21.7	7.48	2	<0.050
	<100,000	24	52.2			
	>100,000	12	26.1			
Bone marrow blasts (%)	<50%	13	28.3	8.70	1	<0.010
	>50%	33	71.7			

Abbreviations: AML, acute myeloid leukemia; ECOG, Eastern Cooperative Oncology Group; CCI, Charlson Comorbidity Index; HCT-CI, Hematopoietic Cell Transplantation Comorbidity Index; LVEF, left ventricular ejection fraction; chi-square test: χ^2^—test value, df—degree of freedom, *p*-value.

**Table 2:** Lines of treatment used and the response obtained.

Variable		Number of Patients	Percentage (%)	Chi-Square Test of Proportion		
				χ^2^	df	*p*
First line	Yes	46	100	--	--	--
	CR	25	54.3	11.20	2	<0.010
	CRi	0	0			
	PR	1	2.2			
	No response	13	28.3			
	Death	7	15.2			
Second line	Yes	32	69.6	7.04	1	<0.010
	CR	9	30.0	1.36	2	0.507
	CRi	1	3.3			
	PR	1	3.3			
	No response	12	40.0			
	Death	7	23.3			
Third line	Yes	15	32.6	5.57	1	<0.050
	CR	3	20	1.60	2	0.449
	Cri	0	0			
	PR	0	0			
	No response	5	33.3			
	Death	7	46.7			
First disease relapse		21/37	59.5	1.00	1	0.317
	<6 mo	14	66.7	2.33	1	0.127
	6–12 mo	7	33.3			
Second disease relapse		8/32	25.0	9.53	1	<0.010
	<6 mo	5	62.5	0.50	1	0.480
	6–12 mo	3	37.5			
Overall survival	<6 mo	20	43.5	2.65	2	0.266
	6–12 mo	11	23.9			
	>12 mo	15	32.6			

Abbreviations: CR, complete remission; CRi, complete remission with incomplete count recovery; PR, partial remission; mo, months; chi-square test: χ^2^-test value; df—degree of freedom; *p*-value.

**Table 3:** Complications, current status, and the cause of death.

Variable		Number of Patients	Percentage (%)	Chi-Square Test of Proportion		
				χ^2^	df	*p*
Complications	Cardiovascular	26	56.5	0.78	1	0.376
	Hematological	46	100	--	--	--
	Hemorrhagic	28	60.9	2.17	1	0.140
	Thrombotic	6	13.0	25.13	1	<0.001
	Bacterial infections	40	87.0	25.13	1	<0.001
	Fungal infections	2	4.3	38.35	1	<0.001
	COVID infection	13	28.3	8.70	1	<0.010
Current status	Deceased	37	80.4	17.04	1	<0.001
	Alive	9	19.6			
	Lost from record	0	0			
Cause of death	Cardiovascular	5	13.9	14.00	4	<0.010
	Hemorrhagic	11	30.6			
	Thrombotic	3	8.3			
	Infectious	14	38.9			
	COVID	3	8.3			
Death < 60 days		9	19.6	--	--	--

Chi-square test: χ^2^—test value; df—degree of freedom; *p*-value.

**Table 4:** Kaplan–Meier analysis results for OS.

	Median				Log-Rank (Mantel–Cox)		
	Estimate	Std. Error	95% Confidence Interval				
			Lower Bound	Upper Bound	χ^2^	df	*p*
Overall	8.00	1.80	4.47	11.53			
RC Line 1							
No	4.00	2.29	0.0	8.49	9.89	1	0.002
Yes	12.00	3.55	5.04	18.96			
RC Line 1 duration							
<6 mo	6.00	1.30	3.45	8.55	5.70	1	0.017
≥6 mo	18.00	11.86	0.0	41.24			
ECOG							
0–1	8.00	1.60	4.86	11.14	14.33	1	0.001
2–4	0.0						
HCT-CI							
0–2	11.00	1.34	8.38	13.63	0.85	1	0.357
3– ≥4	6.00	1.28	3.50	8.50			
CCI							
4	8.00	3.01	2.11	13.89	0.04	1	0.851
≥4	7.00	2.60	2.00	12.03			
AML classification							
De novo	9.00	2.53	4.04	13.96	0.48	1	0.487
Secondary	6.00	2.19	1.71	10.29			
Risk stratification for cytogenetic test							
Favorable	11.00	5.14	0.92	21.08	1.60	1	0.205
Intermediate–adverse	4.00						
Risk stratification for molecular biology test							
Favorable	12.000	4.267	3.637	20.363	8.32	1	0.004
Intermediate–adverse	6.000	1.248	3.554	8.446			
Bone marrow blasts (%)							
<50%	7.00	2.27	2.56	11.44	0.53	1	0.465
≥50%	8.00	1.91	4.25	11.75			

Abbreviations: mo, months; chi-square test: χ^2^—test value; df—degree of freedom; *p*-value.

**Table 5:** Cox regression analysis results.

	Omnibus Test of Model			B	SE	Wald	df	Sig.	Exp(B)	95.0% CI for Exp(B)	
	χ^2^	df	*p*							Lower	Upper
CR after first line of treatment	5.26	1	0.022	−1.22	0.56	4.81	1	0.028	0.30	0.10	0.88
HCT-CI (0–2)											
Risk stratification for cytogenetics (intermediate–adverse)	--	--	--	--	--	--	--	--	--	--	--
Risk stratification for molecular biology (intermediate–adverse)	4.14	1	0.042	1.50	0.74	4.14	1	0.042	4.46	1.06	18.88
HCT-CI (3–≥4)											
Risk stratification for cytogenetics (intermediate–adverse)	0.28	1	0.595	0.35	0.65	0.29	1	0.590	1.42	0.40	5.07
Risk stratification for molecular biology (intermediate–adverse)	2.52	1	0.112	0.78	0.48	2.65	1	0.104	2.18	0.85	5.55
CCI (≥4)											
Risk stratification for cytogenetics (intermediate–adverse)	1.24	1	0.265	0.72	0.62	1.36	1	0.243	2.05	0.61	6.86
Risk stratification for molecular biology (intermediate–adverse)	5.36	1	0.021	1.00	0.42	5.63	1	0.018	2.73	1.19	6.24

Abbreviations: CR, complete remission; chi-square test: χ^2^—test value; df—degree of freedom; *p*-value.

## Data Availability

The original contributions presented in this study are included in the article. Further inquiries can be directed to the corresponding author.
